# A Comparison of Two Methods of Dental Age Estimation in a Population of Saudi Children and Adolescents

**DOI:** 10.3390/diagnostics14171935

**Published:** 2024-09-02

**Authors:** Heba H. Bakhsh, Nada A. Al-shehri, Alanoud Shahwan, Rabab Altuwairqi, Faten J. Mojaleed, Ghaida Alwaalan, Shahad Asaad

**Affiliations:** 1 Department of Preventive Dental Sciences, College of Dentistry, Princess Nourah bint Abdulrahman University, P.O. Box 84428, Riyadh 11671, Saudi Arabia; hhbakhsh@pnu.edu.sa; 2 General Dentist, Private Clinic, Riyadh 13241, Saudi Arabia; alanoud.shahwan@gmail.com (A.S.); g.alwaalan@gmail.com (G.A.); 3 Department of Orthodontics, King Saud Medical City, Riyadh 12746, Saudi Arabia; rtoergi@ksmc.med.sa; 4 Riyadh Second Health Cluster, Ministry of Health, Riyadh 13324, Saudi Arabia; fmojaleed@kfmc.med.sa; 5 Pediatric Resident-Ministry of Health, Riyadh 12211, Saudi Arabia; shahad.asaad1@hotmail.com

**Keywords:** age estimation, Demirjian, London atlas, orthopantomogram, panoramic radiograph, dental age

## Abstract

This study aimed to compare and evaluate the accuracy of the Demirjian (DE) and the London Atlas (LAE) dental age estimation methods in a Saudi population sample. This retrospective cross-sectional study used digital radiographs from electronic health records in three different dental institutes. In total, 357 male and 354 female (ages 5–15 years) digital orthopantomograms were selected for age estimation. The mean difference between the chronological age (CA) and age estimation method among males and females was 0.03 ± 0.34 and 0.00 ± 0.34, respectively, for LAE and 0.55 ± 0.84 and 0.76 ± 0.51, respectively, for DE. The mean difference between the LAE and DE methods among males and females was 0.52 ± 0.89 and −0.76 ± 0.57, respectively. No statistically significant difference between CA and LAE was found in either males (*p* = 0.079) or females (*p* = 0.872). A statistically significant difference was found between CA and DE in both genders (*p* < 0.001). A statistically significant difference was found between the LAE and DE groups (*p* < 0.001) in both genders. An overestimation of dental age was observed with DE compared with that in CA. LAE showed higher accuracy than CA, with no clinically significant difference. Although the difference between the LAE and DE methods was insignificant, the LAE method proved to be more accurate.

## 1. Introduction

Chronological age (CA) is the period of time an individual has lived. CA is important in most societies for legal reasons such as school attendance, employment, social benefits, and marriage [1]. Estimating one’s age is an important component of forensic science for identifying subjects and narrowing the search possibilities for unidentified deceased or living individuals for medico-legal purposes [2]. Dental age estimation can play a crucial role in chronological age investigation by assessing the developmental stage of an individual’s dentition [1,2]. This could hold a special significance in forensic practices, such as in identifying unregistered children and any potential reunification process [1,2].

Moreover, age estimation is important in pediatric endocrinology, archeology, and clinical dentistry [3]. Pediatric and orthodontic dentistry can help guide the treatment process and the need for interceptive interventions [4]. Many anthropologic characteristics, such as the skeletal system, body mass, sexual characteristics, and dental systems, can be used to study an individual’s biological age [3]. Several techniques, including visual assessment of tooth eruption and tooth development and maturation methods, have been suggested for determining CA based on dental characteristics [5,6]; however, visual assessment of tooth eruption provides a smaller window of age estimation, as teeth erupt over a short period in life [6]. It has also been shown to be affected by a multitude of environmental factors, including dental arch space, early extraction of primary teeth, tooth impaction, and tipping [5]; therefore, age estimation using the developmental stage of a tooth is more useful because it depends on tooth calcification, which can be assessed over a long period of time with the help of radiographs, leading to more accurate and reliable results [5,6]. The CA estimated from the tooth developmental stage can be assessed by evaluating clinically useful radiographic images, especially dental orthopantomograms (OPGs), which show the entire dentition in a single image. An OPG provides clinical investigators with a uniquely effective way of assessing the timing of eruption and the degree of classification of developing teeth [6]. Age estimation through dental maturation has been performed by multiple authors who have developed scoring methods, including Demirjian and Goldstein (1973) [7] and Nolla (1960) [8]. Demirjian et al. published the most commonly tested method for dental age (DA) assessment, which had potential because of its feasibility and accuracy [9]. This method was first developed in 1973 using a sample of French–Canadian children [7] and is used to evaluate the development of seven mandibular teeth from an OPG to calculate the dental age [7]. A new age estimation method, The London Atlas of Human Tooth Development and Eruption, was developed by ALQahtani et al. in 2010 [10]. This method uses a pictorial book that requires the investigator to assess the stage of formation and eruption of each tooth and then match it to 1 of 31 illustrations of age categories representing both tooth formation and tooth eruption [10]. A software version of the atlas was developed, allowing rapid age estimation by trained investigators, showing a higher potential for accuracy [10,11]. The accuracy of the dental age estimation methods has been evaluated in multiple populations [9]. Various age assessment methods have shown high degrees of reliability in different populations. However, other studies found that the results were inaccurate when applied to certain populations, as the method either overestimates or underestimates CA [12].

In Saudi Arabia, various studies have estimated the age of children and adolescents using the Demirjian age estimation method. A study conducted in Jeddah revealed a significant correlation between chronological and dental ages in both genders [13]; however, a separate study conducted in Riyadh in patients aged 9–14 years revealed that dental age was advanced compared to the CA [14]. Many previous studies have been conducted with small sample sizes. Notably, only a few studies have compared the Demirjian and London Atlas methods in Saudi Arabia. Hence, this study aimed to investigate the accuracy and precision of dental age estimation in Saudi children and adolescents using the Demirjian and London Atlas methods. This study provides insights into the method that is more accurate and can be used to improve dental age estimation in the Saudi population.

## 2. Materials and Methods

### 2.1. Sample Selection

This retrospective cross-sectional study used available digital OPG radiographs obtained during the last seven years from patients’ electronic health records for diagnostic reasons. Digital OPG radiographs were obtained from the archives of three different dental institutes: Princess Nourah bint Abdulrahman University, King Saud Medical City, and the Riyadh Specialized Dental Center. A database of 1238 OPG radiographic images chosen randomly from dental patient files from the past seven years was collated from all three centers. The radiographs were divided into 11 age groups, ranging from 5 to 15 years for males and females. Images that met the inclusion criteria below for each age group were included. Of the collected 1238 radiographs, 711 were included in the study, ensuring at least 30 radiographs per age group for both males and females. Each OPG was de-identified, coded, and stored as a JPEG image on a hard drive. Ethical approval was obtained from the Institutional Review Board of Princess Nourah bint Abdulrahman University (PNU), H-01-R-059, and Ministry of Health 2019-0172E.

For the OPG radiograph to be included, the patient had to meet all the following criteria: is a Saudi national, was between the ages of 5 and 15 years on the day the X-ray was taken, had no developmental or systemic disorders according to the medical records, and all permanent teeth, except third molars, were present in both dentitions of the lower left side. Finally, only images with adequate exposure, optimal contrast, adequate density, and accurate depiction of the dental and skeletal structures were included.

On the other hand, X-rays of patients with systemic diseases or genetic disorders that could affect skeletal and dental growth or with the presence of localized oral pathology, anomalies, or impacted teeth that could affect dental growth were excluded. In addition, OPGs that had poor quality, in which one or more targeted teeth could not be scored due to patient positioning or imaging technique, and OPGs for non-Saudi patients were excluded.

### 2.2. Measurements

Three examiners performed age estimation measurements. The inter-examiner reliability was 81% using Cohen’s kappa statistic (*p*-value < 0.001). The samples were randomly divided among the three examiners for measurement, each evaluating 237 OPG radiographs. The digitized OPGs were viewed on a widescreen monitor with Microsoft Office Picture Manager 2010 (Microsoft Corp., Redmond, WA, USA), with the gender of the patient identified. A self-generated Google form was created for the Demirjian Age Estimation (DE) method. The Queen Mary University electronic interactive software [10] was used for the London Atlas Age Estimation (LAE) method and the estimated age was manually entered into a Google form.

### 2.3. Methods of Age Estimation

#### 2.3.1. Demirjian Age Estimation Method

Each tooth from the left lower quadrant, except the third molar, was rated based on the eight stages (from “A” to “H”) of tooth calcification from the tip of the cusp to the closure of the apex, according to the crown formation, pulp chambers, and the root length [7]. For each tooth, the scale of maturity was converted into a score using the criteria described by Demirjian et al. and the total maturity score was obtained as the sum of the scores for all seven teeth. Second, the maturity score was transformed into a DA according to tables designed by Demirjian et al. [7]. If the tooth on one side was difficult to visualize, the contralateral tooth was assessed.

#### 2.3.2. The London Atlas of Human Tooth Development and Eruption

LAE was performed using the Queen Mary University electronic interactive software [10]. The London Atlas (Queen Mary Innovation Ltd., London, UK) is a pictorial book that requires the investigator to assess the stage of formation and eruption of each tooth and then match it to one of 31 illustrations of age categories representing both tooth formation and tooth eruption [10]. The tooth formation stages were adapted from Moorrees et al. [15], and the eruption stages were determined by performing the analysis by Bengtson [16].

#### 2.3.3. Reporting of Age

For both methods, age was reported in years with two decimal spaces from the date of birth throughout the paper. The estimated age was deducted from the CA for each sample to determine the accuracy of the age estimation for each method. The resulting age difference represents an overestimation if the difference is positive and an underestimation if the result is negative. The clinically acceptable range for the estimated age was set to be ±0.25 years from the actual CA of the patient, as suggested by the principal investigators (orthodontist and pediatric dentist) for clinical use.

### 2.4. Statistical Testing

All data were collected, tabulated, and statistically analyzed using SPSS version 20 (IBM Corp., Armonk, NY, USA). All continuous variables were initially tested for normality using the Kolmogorov–Smirnov test, and the data were found to be non-normally distributed; hence, within- and between-group comparisons were performed using nonparametric tests (i.e., Wilcoxon signed-rank test). The data are presented as mean and standard deviation.

## 3. Results

A total of 711 OPGs (357 males and 354 females) aged 5–15 years were selected for the age estimation. The sample distributions for age and gender are presented in Table 1. The mean differences between CA and the estimation methods for both genders are shown in Table 2. The mean difference between CA and LAE among males and females gave a mean value of 0.03 ± 0.34 and 0.00 ± 0.34, respectively. The mean difference between CA and DE among males and females showed a mean value of 0.55 ± 0.84 and 0.76 ± 0.51, respectively. The mean difference between the LAE and the DE methods among males and females gave a mean value of 0.52 ± 0.89 and −0.76 ± 0.57, respectively. The sample did not demonstrate a normal distribution; hence, the non-parametric Wilcoxon signed-rank test was used to assess the statistical significance between the CA and estimated age (EA), with a *p*-value of <0.05 set as statistically significant. For the overall sample (Table 2), no statistically significant differences between CA and LAE were found in males and females (*p* = 0.079 and *p* = 0.872, respectively). Meanwhile, a statistically significant difference was found between CA and DE in both males and females (*p* < 0.001). A statistically significant difference was found between LAE and DE (*p* < 0.001) for both genders.

**Figure 1:**
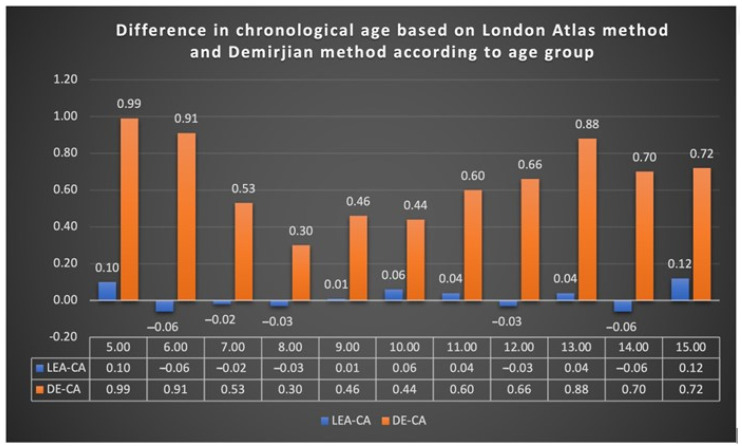
Differences in chronological age based on the London Atlas method and Demirjian method according to age group.

When comparing the mean differences between the age estimation methods and CA for each age group for males (Table 3) and females (Table 4), no statistically significant difference was found between CA and LAE in both males and females (*p* < 0.05), except for the female age groups of 5 years (*p* = 0.010), 12 years (*p* = 0.044), and 15 years (*p* = 0.025). There was a statistically significant overestimation in the female age groups of 5 and 15 years and an underestimation in the female age group of 12 years (Figure 1); however, the difference was not clinically significant as it did not exceed the allowed 0.25-year limit for age estimation error set in the study. A statistically significant difference was found between CA and DE among all age groups for males and females, except for the male age group of 10 years (*p* = 0.443), which showed no statistically significant differences. A statistically significant difference was found between LAE and DE among all age groups for males and females (*p* <0.05), except for the male age groups of 9, 10, and 11 years (*p* > 0.05). Figure 1 shows the differences between the age estimation methods and the CA. An overestimation of dental age was seen with DE compared with CA, while LAE showed more accuracy compared with CA, although with no clinically significant difference.

## 4. Discussion

Many basic aspects of an individual’s identity revolve around name, gender, nationality, and age; however, a significant number of births are unnoticed and unrecorded by authorities [17]. This leads to children having no recognized identity in society, violating the United Nations Convention on the Rights of the Child [17]. An individual’s CA is defined as the amount of time they have existed [1]. There are multiple methods for age estimation, including physical examinations, radiographic examinations of the hand bones, and dental examinations using OPG radiographs, among others [18]. In the field of clinical dentistry, age can play a critical role in orthodontic diagnosis and treatment planning, especially in a growing child [4]. Dental age provides a deeper insight into teeth development and eruption status, which are significant factors in pedodontics and orthodontic treatment. Based on a child’s maturity and level of dental development, a decision can be made about the need for teeth extraction, space management, and the type and timing of orthodontic intervention [1], as chronological and dental age might not be consistent in all individuals [4,13]. In this study, a sample of 711 OPGs from Saudi children and adolescents divided into roughly equal numbers in each age group (5–15 years) for both genders was used to assess the accuracy of the LAE and DE methods.

The findings of this study demonstrate that when using the LAE method on a sample of the Saudi population, the mean difference between the estimated and chronological ages was only 0.03 ± 0.34 years for males and 0.00 ± 0.34 years for females. Although there was a statistically significant overestimation in the female age groups of 5 and 15 years and an underestimation in the female age group of 12 years, these findings were not clinically significant, as they did not exceed the proposed allowed 0.25-year limit of error in age estimation for clinical use. This study showed a strong association between CA and dental age when LAE was used in both genders. Similar results were found in a 2015 study by Alshihri et al. using the LAE method, with 65.5% of the participants having age estimations within 12 months of their CA. Additionally, 19.0% revealed an overestimated CA and 15.5% revealed an underestimated CA by >12 months [12]. Alshihri et al. used a 12-month window estimation, whereas, in this study, the estimation outcome was considered clinically acceptable when it was within 3 months of the CA. The discrepancy between our findings and those of Alshihri et al. may be explained by the diversity in ethnicity, culture, and environment within the Jeddah population sample. Alshihri et al. also had a smaller sample size (*n* = 252) and a non-uniform sample distribution among different male and female groups. Alsudairi and AlQahtani (2019) examined two age estimation methods: Cameriere’s formula and the LAE method [19], with the LAE method showing a mean difference in CA and estimated age of −0.59 years ± 1.45 years. Notably, they demonstrated an underestimation of CA when using the LAE method. While Alsudairi and AlQahtani (2019) indicated an underestimation, this study revealed the accuracy of age estimation when employing LAE. These discrepancies in the findings of the mean difference between LAE and CA could be explained by the smaller sample size used by Alsudairi and AlQahtani (2019) (*n* = 400).

The DE method is a practical and widespread method for estimating dental age and has been used in numerous studies on different populations, with variable outcomes [6]. Some investigators observed a similar pattern of accuracy with the population they studied [13], while others showed either an overestimation [20,21,22] or an underestimation [23] of dental age in relation to CA. In the 5–15-year-old age groups evaluated in this study, the estimated dental age overestimated the CA in both males and females when using DE. In the total sample of 711 children and adolescents, the mean difference between the dental and chronological ages was 0.55 ± 0.84 years in males and 0.76 ± 0.51 years in females. These differences were statistically significant (*p* < 0.05). The overestimation demonstrated in our study sample was also found in other studies in Saudi Arabia—between 0.3 and 0.85 years for males and between 0.4 and 0.85 years for females [21,24].

According to Al Emran et al. (2008), the dental age of Saudi children between 8.5 and 17 years old was overestimated by 0.3 years in males and 0.4 years in females when using the Demirjian method [21]. In a later study in 2013, Baghdadi examined Saudi children between the ages of 4 and 14 using the same method, discovering a mean difference of 0.77 ± 0.85 years for males and 0.85 ± 0.79 years for females [24].

According to a study by Alotaibi (2023) on Saudi children, all six dental age estimation methods revealed a substantial discrepancy between dental age and CA, but the Demirjian approach showed the greatest level of accuracy, with a mean difference of 0.15 years [25]. Another study by Qudeimat (2009) conducted on Kuwaiti children aged 3–14 years found an overestimation, with a mean difference of 0.71 ± 1.18 years in males and 0.67 ± 1.30 years in females [26]; moreover, a study by Shen et al. (2022) in China showed a 0.647-year overestimation of dental age using the Demirjian method in a group of 5–13-year-old subjects [27]. Similarly, a study by Galibourg et al. (2021) in a population of 2–24-year-old French subjects showed an overestimation of the age of females by 0.18 years and males by 1.2 years when using the Demirijan method [28].

Al-Dharrab et al. (2017) found that dental age was underestimated in some female age groups and overestimated in some male age groups; however, the correlation analysis showed that the difference was statistically insignificant [13]. Ashraf et al. (2020) also found results that differ from those in this study, demonstrating that the DE method is the most accurate method when compared to the LAE and William methods among Saudi children and adolescents [23].

A study by Gelbrich et al. (2020) compared three dental age estimation methods for forensic age estimation and found a higher level of accuracy for the Willems method than for the Demirjian method, with the London Atlas method showing comparable results. This study recommends the use of the average of the Willems and London Atlas for an accurate age estimation [29].

The overestimation demonstrated in this study and other studies when using the DE method could be explained by the lack of more precise stages that show each different stage of tooth development as seen with the LAE method. One of the main disadvantages of the DE method is that its stages are not as detailed as those of the LAE method, making calculation and estimation difficult. Another disadvantage is the difficulty in calculating scores for missing teeth. On the other hand, the LAE method showed a more precise age estimation and more accessible use in this study sample.

In addition, evaluation consistency, time efficiency, and ease of use were several reasons why the LAE method was superior to the linear DE method; additionally, teeth on both sides of the mouth can be used for LAE rather than teeth on the left lower jaw alone, as used in Demirjian’s technique. Finally, the availability of an extra dental developmental stage in the LAE method, which details the apex closure scores, resulted in better measures of accuracy.

This study was undertaken to determine the accuracy of the DE and LAE methods when applied to the Saudi Arabian population. The sample was collected from different government clinics and university centers in Riyadh, where patients come from different regions of the country to receive general and specialized dental treatment. This makes the sample more representative of the Saudi population, unlike other studies performed on the Saudi population from only one center. Other strengths of this study are the sample size and distribution among different age groups. The authors ensured the collection of a broad and adequate sample of radiographs while ensuring the quality of radiographs per group.

This study has some limitations. This was a retrospective study of OPGs sampled from the patient record database of a teaching hospital and a governmental dental clinic. While Riyadh is the most populated region in the country, with Saudi nationals from all over the country, the results from this sample cannot be generalized with certainty to the Saudi population. Second, the quality of the X-rays used for the enrolled sample of children’s age range (5–7 years) could have been affected by the artifacts produced by the difficulty in positioning patients and the unwanted movement of patients in this age group, affecting the accuracy of age estimation.

We suggest that future studies be conducted on a more varied population from numerous regions of Saudi Arabia to make the results more generalizable. Researchers from different regions can collaborate to gather larger samples from all over the country. In this study, the researchers developed a comprehensive online form for the DE method to perform age estimation with an average time of 5 min per image compared to the 10–12 min required for the original DE method. Other researchers could take this approach further and implement artificial intelligence through deep learning for age estimation from dental radiographs. This could ease the process for practitioners by automating and easily producing population-specific modifications if needed.

## 5. Conclusions

When evaluating the accuracy of the DE and LAE age estimation techniques for a Saudi population aged between 5 and 15 years, LAE proved to be more accurate. Only the female age groups of 5 and 15 years showed a statistically significant overestimation, while the female age group of 12 years showed an underestimation, although the difference was clinically insignificant. In contrast, the DE method showed a considerable difference between the estimated ages and patients’ CAs, with a consistent overestimation that was statistically significant and considerably higher than the clinically significant level.

## Figures and Tables

**Table 1:** Distribution of study participants based on gender and age.

Age Group	Age Range	Male		Female		Mean	Std. Deviation
		N	%	N	%		
5	4.95–<5.95	30	8.1	32	8.8	5.40	0.33
6	5.95–<6.95	39	10.6	30	8.3	6.39	0.31
7	6.95–<7.95	30	8.1	34	9.4	7.38	0.30
8	7.95–<8.95	32	8.7	30	8.3	8.41	0.30
9	8.95–<9.95	30	8.1	35	9.7	9.38	0.31
10	9.95–<10.95	31	8.4	32	8.8	10.39	0.28
11	10.95–<11.95	37	10.0	30	8.3	11.38	0.27
12	11.95–<12.95	30	8.1	36	9.9	12.47	0.30
13	12.95–<13.95	33	8.9	30	8.3	13.38	0.29
14	13.95–<14.95	30	8.1	35	9.7	14.40	0.29
15	14.95–<15.95	35	9.5	30	8.3	15.34	0.29
5–15 years	4.95–<15.95	357	50.21	354	49.78	10.40	3.17

**Table 2:** Comparison of chronological age with age estimated using London estimation and Demirjian estimation methods.

Gender		N	Minimum	Maximum	Mean	Std. Deviation	Mean Difference	*p*-Value
Male	London Estimation–Chronological age	357	−0.70	1.76	0.03	0.34	0.03	0.079
	Demirjian Estimation–Chronological age	357	−1.93	2.74	0.55	0.84	0.55	<0.001 ***
	London Estimation–Demirjian Estimation	357	−2.50	2.30	−0.52	0.89	−0.52	<0.001 ***
Female	London Estimation–Chronological age	354	−0.81	0.64	0.00	0.34	−0.002	0.872
	Demirjian Estimation–Chronological age	354	−0.84	3.21	0.76	0.51	0.76	<0.001 ***
	London Estimation–Demirjian Estimation	354	−3.10	0.80	−0.76	0.57	−0.76	<0.001 ***

*** *p* < 0.001 is very highly statistically significant. Wilcoxon signed-rank test.

**Table 3:** Comparison of age estimates given by chronological age, London Atlas, and Demirjian methods according to different age groups in males.

Age (Males)	N	LAE–CA		DE–CA		LAE–DE	
		Mean Diff.	*p*-Value	Mean Diff.	*p*-Value	Mean Diff.	*p*-Value
5	30	0.04	0.523	0.97	<0.001 ***	−0.93	<0.001 ***
6	39	−0.05	0.385	0.95	<0.001 ***	−1.00	<0.001 ***
7	30	−0.03	0.583	0.68	<0.001 ***	−0.71	<0.001 ***
8	32	−0.04	0.496	0.35	0.004 *	−0.39	0.007 *
9	30	0.09	0.137	0.31	0.044 *	−0.22	0.149
10	31	0.13	0.116	−0.11	0.443	0.24	0.157
11	37	0.05	0.327	0.32	0.046 *	−0.26	0.078
12	30	0.05	0.323	0.55	<0.001 ***	−0.49	<0.001 ***
13	33	0.02	0.663	0.85	<0.001 ***	−0.83	<0.001 ***
14	30	−0.02	0.668	0.45	0.030 *	−0.47	0.016 *
15	35	0.11	0.063	0.67	<0.001 ***	−0.56	<0.001 ***

* *p* < 0.05 is statistically significant. *** *p* < 0.001 very highly is statistically significant.

**Table 4:** Comparison of age estimates given by chronological age, London Atlas, and Demirjian methods according to different age groups in females.

Age (Females)	N	LAE–CA		DE–CA		LAE–DE	
		Mean Diff.	*p*-Value	Mean Diff.	*p*-Value	Mean Diff.	*p*-Value
5	32	0.15	0.010 **	1.00	<0.001 *	−0.85	<0.001 *
6	30	−0.08	0.196	0.86	<0.001 *	−0.94	<0.001 *
7	34	0.00	0.991	0.40	<0.001 *	−0.40	<0.001 *
8	30	−0.01	0.824	0.24	0.012 *	−0.26	0.024 *
9	35	−0.06	0.295	0.58	<0.001 *	−0.65	<0.001 *
10	32	−0.01	0.912	0.98	<0.001 *	−0.98	<0.001 *
11	30	0.03	0.557	0.95	<0.001 *	−0.92	<0.001 *
12	36	−0.11	0.044 *	0.75	<0.001 *	−0.86	<0.001 *
13	30	0.06	0.339	0.91	<0.001 *	−0.86	<0.001 *
14	35	−0.09	0.176	0.91	<0.001 *	−1.00	<0.001 *
15	30	0.12	0.025 *	0.77	<0.001 *	−0.65	<0.001 *

* *p* < 0.05 is statistically significant. ** *p* < 0.01 is highly statistically significant.

## Data Availability

The data presented in this study are available on request from the corresponding author. Ethical approval from the data collection centers is required to share radiographs due to ethical agreement restrictions.
